# Search for genes responsible for the remarkably high acetic acid tolerance of a *Zygosaccharomyces bailii*-derived interspecies hybrid strain

**DOI:** 10.1186/s12864-015-2278-6

**Published:** 2015-12-16

**Authors:** Margarida Palma, Filipa de Canaveira Roque, Joana Fernandes Guerreiro, Nuno Pereira Mira, Lise Queiroz, Isabel Sá-Correia

**Affiliations:** Department of Bioengineering, Institute for Bioengineering and Biosciences, Instituto Superior Técnico, Universidade de Lisboa, Av. Rovisco Pais, 1049-001 Lisbon, Portugal

**Keywords:** Yeast, *Zygosaccharomyces bailii*, Acetic acid tolerance genes, Yeast hybrid strains, Food spoilage yeasts, Weak acid food preservatives

## Abstract

**Background:**

*Zygosaccharomyces bailii* is considered the most problematic acidic food spoilage yeast species due to its exceptional capacity to tolerate high concentrations of weak acids used as fungistatic preservatives at low pH. However, the mechanisms underlying its intrinsic remarkable tolerance to weak acids remain poorly understood. The identification of genes and mechanisms involved in *Z. bailii* acetic acid tolerance was on the focus of this study. For this, a genomic library from the highly acetic acid tolerant hybrid strain ISA1307, derived from *Z. bailii* and a closely related species and isolated from a sparkling wine production plant, was screened for acetic acid tolerance genes. This screen was based on the transformation of an acetic acid susceptible *Saccharomyces cerevisiae* mutant deleted for the gene encoding the acetic acid resistance determinant transcription factor Haa1.

**Results:**

The expression of 31 different DNA inserts from ISA1307 strain genome was found to significantly increase the host cell tolerance to acetic acid. The *in silico* analysis of these inserts was facilitated by the recently available genome sequence of this strain. In total, 65 complete or truncated ORFs were identified as putative determinants of acetic acid tolerance and an *S. cerevisiae* gene homologous to most of them was found. These include genes involved in cellular transport and transport routes, protein fate, protein synthesis, amino acid metabolism and transcription. The role of strong candidates in *Z. bailii* and *S. cerevisiae* acetic acid tolerance was confirmed based on homologous and heterologous expression analyses.

**Conclusions:**

ISA1307 genes homologous to *S. cerevisiae* genes *GYP8*, *WSC4*, *PMT1*, *KTR7*, *RKR1*, *TIF3*, *ILV3* and *MSN4* are proposed as strong candidate determinants of acetic acid tolerance. The ORF ZBAI_02295 that contains a functional domain associated to the uncharacterised integral membrane proteins of unknown function of the DUP family is also suggested as a relevant tolerance determinant. The genes *ZbMSN4* and *ZbTIF3*, encoding a putative stress response transcription factor and a putative translation initiation factor, were confirmed as determinants of acetic acid tolerance in both *Z. bailii* and *S. cerevisiae*. This study provides valuable indications on the cellular components, pathways and processes to be targeted in order to control food spoilage by the highly acetic acid tolerant *Z. bailii* and *Z. bailii*-derived strains. Additionally, this information is essential to guide the improvement of yeast cells robustness against acetic acid if the objective is their use as cell factories.

**Electronic supplementary material:**

The online version of this article (doi:10.1186/s12864-015-2278-6) contains supplementary material, which is available to authorized users.

## Background

*Zygosaccharomyces bailii* is considered the most problematic spoilage yeast found in the food and beverage industry, particularly in acidic foods, soft drinks, fruit juices, dairy products and salad dressings [1, 2]. This yeast species ability to cause spoilage derives from its outstanding intrinsic capacity to resist to weak acids widely used as fungistatic preservatives, such as acetic, propionic, benzoic and sorbic acids [1–4]. Understanding the mechanisms of weak acid resistance is central to the development and implementation of more effective food and beverage preservation strategies in order to minimise economic losses. Although *Z. bailii* is the spoilage yeast that exhibits the highest level of tolerance to acetic acid, most of the scientific contributions on the mechanisms underlying adaptation and resistance to acetic acid in yeast have been focused on the more susceptible experimental model *Saccharomyces cerevisiae* [5–9]. At a pH equal or below its p*K*a (4.75), acetic acid is mainly in the undissociated form that can diffuse across the plasma membrane. Once in the near neutral cytosol, acetic acid dissociates leading to the accumulation of protons and acetate. The acidification of the cytosol leads to the inhibition of metabolic activity and to the dissipation of the proton gradient across plasma membrane required for secondary transport [10, 11]. To counteract this effect, the plasma membrane proton-pumping ATPase (PM H^+^-ATPase) is activated in acetic acid-stressed *S. cerevisiae* cells [12]. The involvement of *Z. bailii* PM H^+^-ATPase in the active export of protons from cells challenged by weak acid preservatives, namely benzoic acid, was also demonstrated, suggesting that both yeasts share this response mechanism [13]. Given that the charged acetate counterion is not able to easily cross the hydrophobic plasma membrane lipid bilayer, it accumulates in the cell interior leading to increased oxidative stress and turgor pressure, among other effects [3, 9]. To counteract these effects, the plasma membrane multidrug resistance (MDR) transporters of the Major Facilitator Superfamily (MFS) Tpo2 and Tpo3 were hypothesized to play a role in the extrusion of acetate from acetic acid-challenged *S. cerevisiae* cells [5]. To date, no acetate export system was described in *Z. bailii. S. cerevisiae* response to acetic acid-induced stress involves several transcriptional regulators [4, 9]. The expression of the transcription factor encoding gene *HAA1* was found to markedly decrease the duration of the adaptation period of a yeast cell population suddenly exposed to toxic concentrations of acetic acid, by decreasing the loss of cell viability that occurs during this phase of growth latency [5]. Haa1 is considered one of the key players in the control of *S. cerevisiae* response to acetic acid due to its role in the direct, or indirect, regulation of approximately 80 % of acetic acid-responsive genes [6], several of them required for maximal tolerance to acetic acid [8]. These genes code for protein kinases, MDR transporters, transcription factors and proteins involved in lipid metabolism and nucleic acid processing [6].

In order to identify determinants of *Z. bailii* tolerance to acetic acid at the genome level we used in this study a genomic library previously prepared from the highly acetic acid tolerant strain ISA1307, an interspecies hybrid between *Z. bailii* and a closely related species which was isolated from a continuous production plant of sparkling wine [14, 15]. This genomic library was used to rescue the high susceptibility phenotype of *S. cerevisiae* BY4741_*haa1*Δ. This mutant, deleted for *HAA1* gene, was chosen due to its very high susceptibility to acetic acid to avoid the use of the much higher concentrations required to inhibit the parental strain growth. With this approach we also expected to identify the functional homologue of *S. cerevisiae HAA1* gene in this hybrid strain. During the development of this study, our laboratory carried out the genome sequencing, assembly and annotation of ISA1307 [14]. This hybrid strain has been on the focus of several physiological studies, some of them aiming at the understanding of the mechanisms underlying its remarkable intrinsic resistance to acetic acid. Differently from *S. cerevisiae*, the *Z.bailii*-derived hybrid strain ISA1307 co-consumes glucose and acetic acid when cultivated in glucose medium supplemented with a sublethal growth inhibitory concentration of acetic acid [16, 17]. Quantitative proteomic studies on the adaptive response of this strain indicate that in glucose and acetic acid cultures the acid is channelled through the TCA cycle [16]. After glucose exhaustion, acetic acid being present as the sole carbon source, the content of several proteins involved in gluconeogenesis and pentose phosphate pathway was however found to increase [16].

The screening of ISA1307 genomic library for genes required for tolerance to acetic acid successfully pointed out several strong candidates, but since this is a hybrid strain difficult to be genetically manipulated, in order to confirm the role of the selected genes in acetic acid tolerance we have explored another strain, *Z. bailii* IST302. This strain was isolated from spontaneous fermentation of wine must and, contrarily to ISA1307 and *Z. bailii* CLIB213^T^ [18], does not flocculate and proved to be more susceptible to genetic engineering than these reference strains. The genome of *Z. bailii* IST302 was recently sequenced and annotated in our laboratory (unpublished data), rendering possible the work here reported.

The knowledge gathered during the present study was based on the expression of ISA1307 genomic library in *S. cerevisiae*, the availability of the genome sequences of the hybrid strain ISA1307 and *Z. bailii* IST302 and the expression of selected ISA1307 and IST302 gene sequences in *S. cerevisiae* and *Z. bailii* strains and led to the identification of a number of *Z. bailii* genes involved in tolerance to acetic acid. Strong candidates for determinants of acetic acid tolerance in *Z. bailii* or *S. cerevisiae* are here proposed.

## Results

### Selection of *S. cerevisiae* transformants with increased tolerance to acetic acid through the expression of an ISA1307 genomic library

**Fig. 1:**
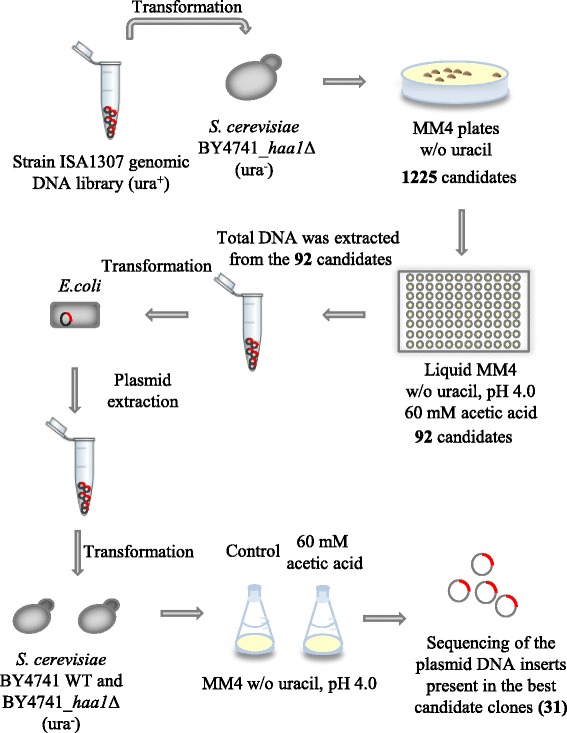
Genomic library screening to search for determinants of acetic acid tolerance in the strain ISA1307. Schematic representation of the screening of the *Z. bailii*-derived interspecies hybrid strain ISA1307 genomic library carried out in this study to search for determinants of acetic acid tolerance, able to rescue the susceptibility phenotype of *S. cerevisiae* BY4741_*haa1*Δ

**Fig. 2:**
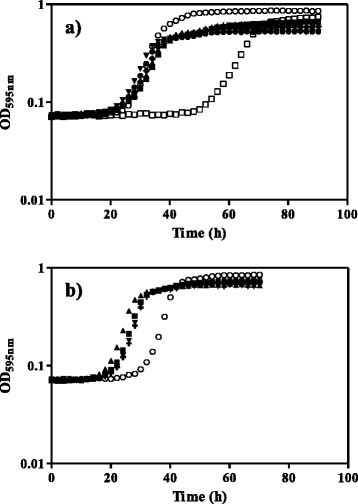
Growth curves of the best strains considering acetic acid tolerance obtained by transformation of *S. cerevisiae* BY4741_*haa1*Δ and BY4741 parental with ISA1307 genomic library plasmids. **a** *S. cerevisiae* BY4741_*haa1*Δ transformed with the empty vector (□) and with plasmids holding DNA fragments from the strain ISA1307 genomic library B02 (■), B18 (▲), L04 (♦), S06 (▼) and X25 (●) cloned in vector pRS316. *S. cerevisiae* BY4741 parental strain transformed with the empty vector (○) was included in the same 96-well microplate as a positive control. **b** *S. cerevisiae* BY4741 parental strain transformed with the empty vector (○) or with plasmids containing DNA fragments from the strain ISA1307 genomic library B02 (■), B18 (▲), S06 (▼), S07 (+) and W08 (×). The selected strains were those with a significant decreased duration of the latency period of the deletion mutant or in the parental strain when compared with the corresponding strain containing the empty vector. The growth curves were performed in MM4 medium (pH 4.0) without uracil, supplemented with 60 mM acetic acid (pH 4.0) and are representative of at least three independent growth assays that gave rise to similar growth curves

To search for genes involved in the remarkable tolerance to acetic acid in a *Z. bailii*-related strain we looked for suppressors of the susceptibility phenotype of *S. cerevisiae* BY4741*_haa1*Δ through the transformation of this strain with a previously constructed genomic library from the highly tolerant *Z. bailii*-derived interspecies hybrid strain ISA1307 [15]. This allowed the isolation of 1225 yeast clones, selected in a minimal medium without uracil (Fig. 1). These yeast transformants were screened for their increased tolerance to acetic acid through growth in liquid medium in 96-well microplates. This selection methodology allowed the identification of 92 potential candidates out of the 1225 positive colonies initially obtained. Total DNA was extracted from each selected transformant and used to transform *Escherichia coli* XL1-Blue in order to isolate the plasmid insert of interest and to guarantee the identification of a single plasmid capable of suppressing *S. cerevisiae haa1*Δ susceptibility phenotype. The plasmids were extracted from *E.coli* transformants, purified and used to transform again the deletion mutant *S. cerevisiae* BY4741_*haa1*∆ and also the parental strain BY4741. Out of these 92 tested transformants, only 31 DNA inserts were confirmed to substantially increase the *haa1*∆ mutant tolerance to acetic acid and were hence sequenced. Both the parental and the deletion mutant *haa1*Δ cells transformed with each one of these 31 plasmids were grown in 96-well microplates with 60 mM of acetic acid supplemented medium or in control conditions in order to confirm the ability of the corresponding inserts to rescue the *haa1*Δ mutant susceptibility phenotype as well as their effect in the growth of the parental strain transformants (Additional file 1: Figure S1). Considering the *haa1*Δ transformants, the suppressors were able to rescue *haa1*Δ susceptibility phenotype by shortening the latency period by at least 20 h when compared with the *haa1*Δ strain transformed with the empty vector. At the concentration of acetic acid tested (60 mM, pH4.0) the protective effect of the expression of the DNA inserts in the candidate clones was not as evident for the parental strain compared with the *haa1*Δ mutant, since the parental strain is much less susceptible than the mutant to acetic acid. Nevertheless, a number of inserts clearly improve the tolerance of the parental strain transformed with the empty vector at the acetic acid concentration tested. Results obtained with the five best suppressors, or in other words those capable of diminishing at a greater extent the duration of the latency period of the parental or the *haa1*Δ strains transformed with the empty vector, are shown in Fig. 2a and b. Three of these suppressors, the inserts B02, B18 and S06, were identified by the transformation of both the parental and the *haa1* deletion mutant strains.

### *In silico* analysis of ISA1307 genomic DNA fragments whose expression was found to be required for increased acetic acid tolerance in *S. cerevisiae*

The *in silico* analysis of the 31 selected genomic DNA fragments from the *Z. bailii*-derived interspecies hybrid strain ISA1307 led to the identification of a number of ORFs which are putative candidate determinants of tolerance to the acid. Since we found that the expression of DNA inserts containing only one putative truncated ORF also led to the increase of *haa1*Δ tolerance to acetic acid, the incomplete ORFs were also considered for further analysis and the presence of conserved functional domains in the identified inserts was investigated. However, the truncated ORFs comprising less than 15 % of the complete sequence, which contained limited portions of conserved domains or no conserved domains, were disregarded at this phase, resulting in the selection of 32 complete and 33 truncated putative candidate ORFs (Additional file 2).

#### ORFs with homology to *S. cerevisiae* genes

For each ISA1307 selected ORF, the predicted *S. cerevisiae* homologous gene and the corresponding functional description were gathered (Additional file 2). References to previous studies in which the deletion of the gene was already described as leading to an increase or decrease of yeast susceptibility to acetic acid or other weak acids are also indicated. Since the strain ISA1307 is an interspecies hybrid between *Z. bailii* and a closely related species [14], Additional file 2 also includes information regarding the nucleotide sequence identity of each ORF present in the different DNA inserts with *Z. bailii* CLIB213^T^ genome [18] to identify the ORFs that likely are from *Z. bailii*. Interestingly, about one half of the selected DNA inserts that were able to rescue the *haa1*∆ mutant susceptibility phenotype to acetic acid is, presumably, derived from the parental *Z. bailii* species (99–100 % identity with CLIB213^T^ genome) while the other half is, apparently, derived from the other yeast species closely related to *Z. bailii* (93–98 % identity with CLIB213^T^ genome).

#### ORFs with no homology with *S. cerevisiae* genes

For 7 out of the 65 ORFs identified amongst the ISA1307 genomic DNA fragments resulting from the screening, no homology with *S. cerevisiae* genes could be found. In order to obtain additional information that could support the elucidation of their possible function and contribution to confer protection against acetic acid in the *S. cerevisiae haa1*∆ mutant, a prediction of conserved functional domains [19] and transmembrane helices (http://www.cbs.dtu.dk/services/TMHMM/) was attempted. The information gathered for these ORFs is summarised in Table 1 that also includes the detected similarities with other organisms (using the PEDANT database). The DNA inserts E13 and S06, each one holding two complete ORFs, were considered to deserve further attention. The ORF ZBAI_09903 from the E13 DNA insert has homology with pyruvate decarboxylases from different organisms and contains a pyrimidine binding domain (PYR) that can be found in many key metabolic enzymes. The DNA insert E13 also contains the ORF ZBAI_09904 whose encoded protein has a domain characteristic of DNA and RNA helicases, but shows weak similarities with yeast helicases. Moreover, the two ORFs present in the insert S06, which was found to be the best suppressor of the acetic acid susceptibility phenotype of mutant *haa1*∆ and among the best five genomic library inserts whose expression led to increased tolerance to acetic acid in the parental strain, encode two putative membrane proteins. Specifically, ZBAI_02295 holds a domain associated with the DUP family of proteins of unknown function that were suggested to be involved in membrane trafficking processes [20] and ZBAI_02296 contains a PRK15313 domain related to autotransport proteins. Both ORFs have homologues in *Z. rouxii*. These observations reinforce the idea that at least one of these two ORFs is a strong candidate to be considered as a determinant of acetic acid tolerance. Given the apparent relevance of the insert S06 and to test the proposed hypothesis, the individual subcloning of these two ORFs was performed.

**Table 1:** Genomic DNA inserts from strain ISA1307 genomic library required for acetic acid tolerance containing the putative ORFs for which weak or no homology with *S. cerevisiae* genes was found. Information about other ORFs present in the same DNA insert is provided, as well as the best blast hits and the predicted functional and transmembrane domains of the corresponding protein

DNA insert	ORF^a^	ORFs in the same insert^a^	Best blast hits	Functional domains	Transmembrane domains
3.13 (4069 bp)	ZBAI_06221 (40 %)	ZBAI_06218 (47 %)	Glutathione S-transferase from Macrophomina phaseolina (charcoal rot fungus) and putative uncharacterized proteins from *Torulaspora delbrueckii* and several fungi.	Domains related with the Glutathione S-transferase family. In particular, the portion which is present in the DNA library fragment possesses a C-terminal alpha helical domain of the GST family.	-
		ZBAI_06219			
		ZBAI_06220			
18.9 (4511 bp)	ZBAI_03403	ZBAI_03401 (28 %)	Two putative uncharacterised proteins, one from *Z. rouxii* and the other from *T. delbrueckii*.	-	-
		ZBAI_03402			
		ZBAI_03404 (30 %)			
E13 (4470 bp)	ZBAI_09903	ZBAI_09904	Putative and described pyruvate decarboxylases from other yeasts and several other different organisms.	Pyrimidine binding domain (PYR) of pyruvate decarboxylase.	-
	ZBAI_09904	ZBAI_09903	Weak similarities with several helicase-like proteins encoded by the Y’ element of subtelomeric regions from *S. cerevisiae* and *S. kudriavzevii*.	Domain characteristic from DNA and RNA helicases.	-
S06 (4413 bp)	ZBAI_02295	ZBAI_02296	Similarities with hypothetical proteins from *Z. rouxii*, most of them being uncharacterised and others sharing some homology with membrane proteins from DUP240 and DUP380 gene families, such as *COS8*, *COS9* and *YHL044W*.	Domain related with DUP family, which consists of several yeast proteins of unknown functions.	1
	ZBAI_02296	ZBAI_02295	Weak similarities with several hypothetical proteins from *Z. rouxii* which present no similarity with proteins from other species.	PRK15313 domain related to autotransport protein MisL (provisional).	4
V14 (2907 bp)	ZBAI_09508	ZBAI_09509 (99 %)	Putative uncharacterised proteins mainly from other yeasts, such as *Z. rouxii*, *T. delbrueckii* and *Lachancea thermotolerans* species, and fungi.	Zinc-finger related domains.	-

^a^Percentage of nucleotides present in the truncated ORFs, compared with the total ORF sequence, is indicated in parentheses

### The ORF ZBAI_02295 is required for tolerance to acetic acid

**Fig. 3:**
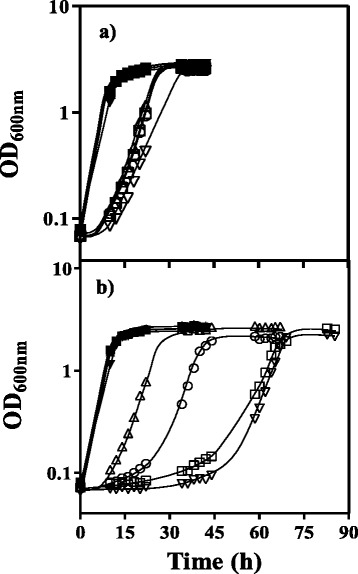
Effect of the expression of ZBAI_02295 or ZBAI_02296 in *S. cerevisiae* parental strain BY4741 and derived deletion mutant *haa1*Δ. Growth curves of *S. cerevisiae* BY4741 parental (**a**) and *haa1*Δ (**b**) strains transformed with pGREG506_ZBAI_02295 (△,▲), pGREG506_ZBAI_02296 (▽,▼), pGREG506_noHIS3 (□,■) and pGREG506 (○,●). Growth was performed in MM4 medium (pH 4.0) without uracil and containing 1 % (w/v) galactose, supplemented with 60 mM acetic acid (open symbols) and in the same conditions without acetic acid (closed symbols). The growth curves are representative of at least three independent growth assays that gave rise to similar results

The two ORFs present in S06 genomic library DNA insert were subcloned by homologous recombination in the expression vector pGREG506 containing a galactose inducible promoter (*GAL1*) and then expressed in both *S. cerevisiae* BY4741_ *haa1*Δ and parental background strains to search for increased tolerance to acetic acid. The heterologous expression of pGREG506_ZBAI_02295 in *haa1*Δ mutant strain was found to lead to a considerable increase of tolerance to acetic acid up to the level of the parental strain with the empty plasmid, with a pronounced decrease in the duration of the latency phase and an increase in the specific growth rate compared with control cells (Fig. 3). This effect was barely detected when the parental strain was used as host cell, even though we have used increased concentrations of acetic acid (65 and 70 mM) in order to obtain an inhibitory effect equivalent to the one observed in the mutant *haa1*Δ (results not shown). On the other hand, the expression of the ORF ZBAI_02296 caused an increase in the duration of the lag phase for both strains (Fig. 3), which indicates that it can be harmful in the context of acetic acid tolerance. For control purposes, Fig. 3 also shows the growth curves of both strains transformed with pGREG506 or pGREG506_noHIS3, that is the same cloning vector but with the *HIS3* gene, controlled by the *GAL1* promoter, removed. This new construction was needed to prepare the correct controls since the recombinant plasmids lose the *HIS3* gene active mark and also because the auxotrophy of the host cells (his^−^) implicates an alteration of the acetic acid tolerance depending on histidine availability. Although in the case of the parental strain the removal of the *HIS3* gene from the expression vector had no detectable implications in the growth curve of the transformed cells, the expression of this gene in *haa1*Δ cells confers a significant advantage when acetic acid is present. Therefore pGREG506_noHIS3 plasmid was considered the correct empty cloning vector to be used to transform the control cells.

The results of the expression experiments support the idea that the ORF ZBAI_02295 should be considered as a strong candidate determinant of tolerance to acetic acid in strain ISA1307. On the contrary, the expression of ORF ZBAI_02296 had a negative effect in *S. cerevisiae* tolerance to acetic acid and for this reason it was not considered as a candidate determinant.

### Selection of strong candidate determinants of acetic acid tolerance

The next step was to narrow down the list of the remaining 63 selected ORFs in an attempt to identify the most promising candidate genes for determinants of tolerance to acetic acid. Although all the selected ORFs are potential candidates, it is essential to understand the relevance of each one when several ORFs are present in the same insert. Due to the difficult genetic engineering of *Zygosaccharomyces* genus in general, and of the ISA1307 hybrid strain in particular, no deletion mutant could be constructed and, consequently, the direct confirmation of the role of each putative candidate ORF in acetic acid tolerance in this specific strain was limited. Therefore, the selection of strong candidate determinants of acetic acid tolerance among the ORFs present in each DNA insert able to rescue the susceptibility phenotype of the *S. cerevisiae haa1*∆ mutant was based on the presumption that sole ORFs present in DNA inserts that were able to suppress the acetic acid susceptibility phenotype of *haa1*∆ are the cause of the increased tolerance. Some of these ORFs homologues were already mentioned as contributing to *S. cerevisiae* tolerance to acetic acid and/or other weak acids, supporting the criterion used. The list of inserts containing the strong candidate determinants of acetic acid tolerance selected during this work is presented in Table 2 (a selection from Additional file 2). The strong candidate determinants of tolerance, according to the mentioned criterion, are represented in bold in both Table 2 and Additional file 2. The ORF ZBAI_02295, whose role in acetic acid tolerance was confirmed by subcloning experiments, is also included. It is important to refer that the expression of truncated ORFs was found to apparently confer tolerance to acetic acid, given that this was observed when a single incomplete ORF was present in the insert. This is for example the case of *S. cerevisiae* truncated *MSN4* and *WSC4* homologues that are present in the DNA inserts B02 and 23.1, respectively. For the inserts where several complete and/or truncated ORFs are present it is not possible, at this phase, to decide which of the various ORFs is indeed involved in conferring increased tolerance to acetic acid without the subcloning and functional expression of each single ORF. Nevertheless, previous reports from the literature on the role of a considerable number of these ORFs in *S. cerevisiae* tolerance to weak acids provide good indications of where to start.

**Table 2:** Genomic DNA inserts from strain ISA1307 genomic library containing the strong candidate determinants of tolerance to acetic acid proposed in this work

DNA insert	ORF^a^	Identity (%) with *Z. bailii* CLIB213^Tb^	*S.cerevisiae* putative homologue	Identity (%) with *S. cerevisiae* ^c^	Size (aa) ISA1307 / *S. cerevisiae* / Alignment^d^	*S.cerevisiae* function^e^	Alteration in yeast deletion mutant susceptibility to weak organic acids^f^
**8.23** (2782 bp)	ZBAI_05695	**99**	***GYP8***	34.0	556 / 497 / 503	GTPase-activating protein involved in the regulation of ER to Golgi vesicle transport**Cellular transport and transport routes**	-
**18.22** (2803 bp)	ZBAI_04770 (41 %)	**95/95**	***PMT1***	66.9	761 / 817 / 746	Protein O-mannosyltransferase	**Acetic acid** (S) [8]
						**Protein fate**	
**23.1** (2277 bp)	ZBAI_09707 (40 %)	**92/95**	***WSC4***	29.1	854 / 605 / 632	ER membrane protein involved in the translocation of soluble secretory proteins and insertion of membrane proteins into the ER membrane; may also have a role in the stress response	-
						**Cellular transport and transport routes**	
**23.17** (1812 bp)	ZBAI_09663 (66 %)	**99/99**	***ILV3***	82.5	583 / 585 / 584	Dihydroxyacid dehydratase, catalyzes third step in the common pathway leading to biosynthesis of branched-chain amino acids	-
						**Amino acid metabolism**	
**B02** (3640 bp)	ZBAI_03527 (77 %)	**95/95**	***MSN4***	32.9	574 / 630 / 590	Transcriptional activator that regulates the general stress response of *S. cerevisiae*	**2,4-D** (S) [41]
						**Transcription**	
**B18** (2917 bp)	ZBAI_01028	**94**	***TIF3***	55.8	433 / 436 / 453	Translation initiation factor eIF-4B	**Acetic acid** (S) [42]**Propionic acid** (S) [7]
						**Protein synthesis**	
**S07** (2860 bp)	ZBAI_05420 (57 %)	**95/95**	***KTR7***	49.3	514 / 517 / 525	Putative mannosyltransferase involved in protein glycosylation	-
						**Protein fate**	
**S06** (4413 bp)	**ZBAI_02295**	**99**	***-***	-	178 / - / -	-	-
	ZBAI_02296	**98**	***-***	-	261 / - / -	-	-
**Y08** (2104 bp)	ZBAI_01926 (34 %)	**92/93**	***RKR1***	48.8	1555 / 1562 / 1571	RING domain E3 ubiquitin ligase; involved in the ubiquitin-mediated degradation of non-stop proteins	-
						**Protein fate**	

^a^Percentage of nucleotides present in the truncated ORFs, compared with the total ORF sequence, is indicated in parentheses

^b^Identity (%) between each ORF found in the DNA inserts and *Z. bailii* CLIB213^T^ genome was obtained using BLAST analysis (http://blast.ncbi.nlm.nih.gov/Blast.cgi)

^c^Identity (%) between ISA1307 and *S. cerevisiae* S288C homologous genes was retrieved from PEDANT database (http://pedant.helmholtz-muenchen.de/genomes.jsp?Category=fungal)

^d^Identity (%) between homologous proteins from ISA1307 and *S. cerevisiae*. The size of the proteins and their pairwise alignment, given by the number of amino acid residues (aa), was retrieved from the PEDANT database (http://pedant.helmholtz-muenchen.de/genomes.jsp?Category=fungal)

^e^The putative function of each ORF was assigned based on the function of each *S. cerevisiae* homologous gene (www.yeastgenome.org). The functional category is provided in bold

^f^List of the studies involving the *S. cerevisiae* corresponding deletion mutant susceptibility or resistance phenotypes under weak acid stress (S) - the single deletion mutant is susceptible to the acid; (R) - the single deletion mutant is resistant to the acid

In summary, and taking into account the aforementioned criterion, the following *S. cerevisiae* gene homologues were selected and proposed herein as strong candidate determinants of acetic acid tolerance in the strain ISA1307: *GYP8* and *WSC4* (cellular transport and transport routes), *PMT1*, *KTR7* and *RKR1* (protein fate), *TIF3* (protein synthesis), *ILV3* (amino acid metabolism) and *MSN4* (transcription).

### Complementation of acetic acid susceptibility of *S. cerevisiae* mutants deleted for genes homologous to ISA1307 tolerance candidate genes

**Fig. 4:**
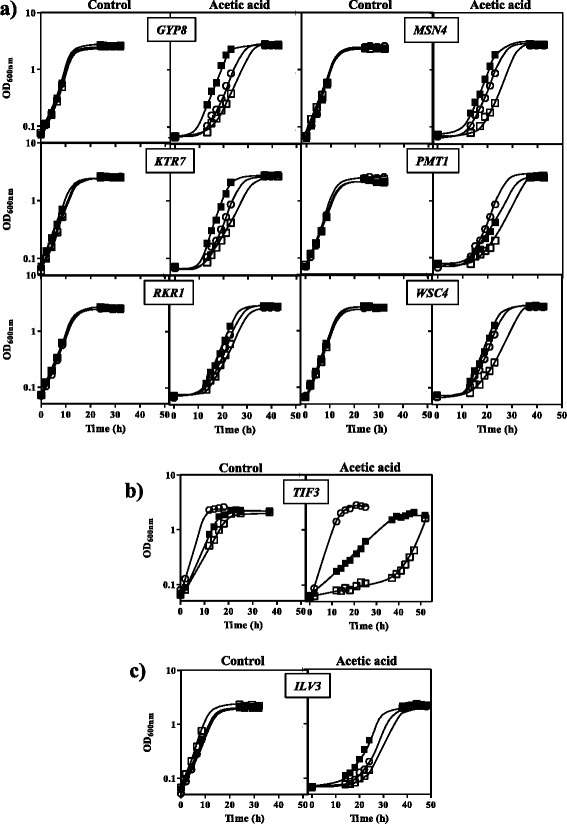
Expression of ISA1307 genomic library plasmids containing a single ORF in *S. cerevisiae* mutants deleted for the corresponding homologous gene. Growth curves of *S. cerevisiae* mutants *gyp8*Δ, *ktr7*Δ, *rkr1*Δ, *msn4*Δ, *pmt1*Δ, *wsc4*Δ, *tif3*Δ and *ilv3*Δ transformed with the corresponding plasmid from the strain ISA1307 genomic library, specifically 8.23, S07, Y08, B02, 18.22, 23.1, B18 and 23.17, respectively, which contained the putative ISA1307 homologue of the *S. cerevisiae* gene deleted in each mutant (■) or with the empty vector (□). Strains were cultivated in MM4 medium (pH 4.0) without uracil or in this same basal medium supplemented with 60 mM (**a**), 40 mM (**b**) and 65 mM (**c**) of acetic acid. *S. cerevisiae* BY4741 and BY4743 parental strains were also transformed with the empty vector (○) for control purposes. The growth curves shown are representative of three independent assays

In order to confirm the hypothesized role of the strong candidate determinants of acetic acid tolerance proposed, plasmids from ISA1307 genomic library containing a single complete or incomplete ORF were expressed in the haploid *S. cerevisiae* mutant lacking the corresponding homologous gene (Fig. 4). The expression of the library plasmids containing *GYP8*, *KTR7*, *MSN4* or *WSC4* homologues was confirmed to rescue the acetic acid susceptibility phenotype of the corresponding *S. cerevisiae* deletion mutant. Specifically, an increase of the maximum specific growth rate was clearly observed when these library plasmids were individually expressed, as well as a reduction of the duration of the latency period even when compared with the parental strain carrying the empty plasmid. Although the expression of the *TIF3* homologue in the corresponding library plasmid also decreased the duration of the latency period in *S. cerevisiae tif3*∆, it did not fully complement the high acetic acid susceptibility phenotype of *tif3*Δ up to the parental strain level. Since at the concentration of acetic acid used in the complementation experiments *S. cerevisiae* BY4741_*rkr1*Δ is mildly susceptible to acetic acid, the effect of the expression in this mutant of the *RKR1* homologue in the duration of the latency phase in the presence of acetic acid was barely detected. The expression of the library plasmid containing the essential *ILV3* homologous gene in the diploid strain *S. cerevisiae* BY4743 lacking a copy of *ILV3* gene also led to the decrease of the duration of the latency phase of both *ilv3*Δ and parental strains. In the case of *pmt1*Δ, a slight complementation of the susceptibility phenotype was achieved with the expression of the corresponding genomic library plasmid.

As previously mentioned, when expressed in the yeast host cell the inserts containing *MSN4* and *TIF3* homologues were among the best suppressors of the acetic acid susceptibility phenotype of *haa1*∆ mutant, also leading to a remarkable increase of acetic acid tolerance in the parental strain. Given that the role of these genes in the context of *S. cerevisiae* tolerance to weak acids was already reported in literature, they were selected for further experiments.

### Expression of *Z. bailii* genes *ZbMSN4* and *ZbTIF3* in *Z. bailii* and *S. cerevisiae* strains

**Fig. 5:**
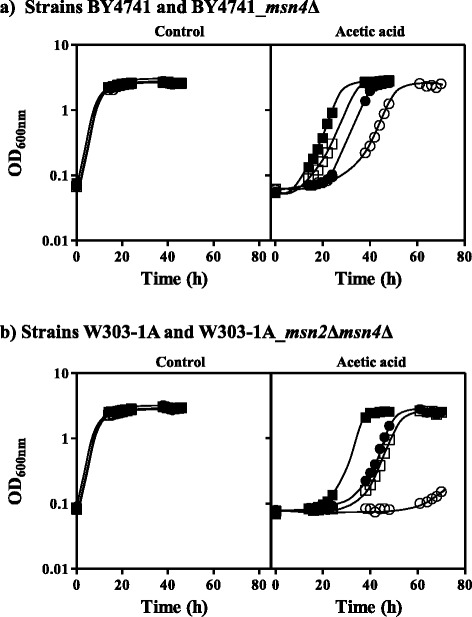
Effect of the expression of *ZbMSN4* in *S. cerevisiae* strains BY4741 parental and derived deletion mutant *msn4*Δ (**a**) and W303-1A parental and derived double deletion mutant *msn2*Δ*msn4*Δ (**b**). Growth curves of *S. cerevisiae* parental strains BY4741 (**a**) and W303-1A (**b**) (*filled symbols*) and the corresponding derived deletion mutants *msn4*Δ (**a**) and *msn2*∆*msn4*Δ (**b**) (*empty symbols*) transformed either with the empty vector (○,●) or with pGREG506_promMSN4Sc_ZbMSN4 (□,■). *S. cerevisiae* BY4741 background strains were cultivated in MM4 medium (pH 4.0) without uracil or in this same basal medium supplemented with 65 mM (**a**), and *S. cerevisiae* W303-1A background strains were cultivated in MM5 medium (pH 4.0) without uracil or in this same basal medium supplemented with 60 mM (**b**). The growth curves shown are representative of three independent assays

**Fig. 6:**
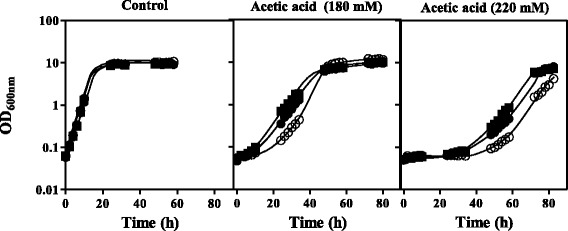
Effect of the expression of an extra copy of *Z. bailii* IST302 genes *ZbMSN4* or *ZbTIF3* in this same strain. Growth curves of *Z. bailii* IST302 transformed either with the empty plasmid pZ_3_ *b*T (○), pZ_3_ *b*T_ *ZbMSN4* (●) or pZ_3_ *b*T_ *ZbTIF3* (■). Yeast cells were cultivated in MM medium, pH4.0, either or not supplemented with acetic acid (180 mM or 220 mM). The growth curves shown are representative of three independent assays

The involvement of *Z. bailii MSN4* homologue in acetic acid tolerance was investigated based on the expression of this gene in *S. cerevisiae* BY4741 parental and derived deletion mutant *msn4*Δ, and in *S. cerevisiae* W303-1A parental and derived double deletion mutant *msn2*∆*msn4*∆ strains (Fig. 5). Since ISA1307 hybrid genome contains two copies of the *MSN4* homologue that have similar 5′ and 3′ ends, it would be very difficult to amplify only the desired *Z. bailii* copy and for this reason strain *Z. bailii* IST302 was used to amplify the *MSN4* homologue *ZbMSN4*. Results of this heterologous expression show that *ZbMSN4* under the control of *S. cerevisiae MSN4* promoter increases the tolerance of the parental *S. cerevisiae* strains BY4741 and W303-1A and derived deletion mutant strains *msn4*Δ and *msn2*Δ*msn4*∆. While in W303-1A_*msn2*∆*msn4*Δ strain the expression of *ZbMSN4* nearly complemented the acetic acid susceptibility phenotype to the level of the parental strain, in BY4741_*msn4*Δ the expression of the gene significantly improved the acetic acid tolerance even above the level of tolerance exhibited by its parental strain, as in the case of the complementation with the library plasmid B02. These results show that IST302 *ZbMSN4* under the influence of *S. cerevisiae MSN4* promoter in the selected expression vector overcomes the effect of the native *S. cerevisiae MSN4* and is effective enough to counteract the lack of the two transcription factors in the double deletion mutant. Extra copies of *ZbMSN4* and *ZbTIF3* from *Z. bailii* IST302 were also expressed in this same strain. Results confirm that *Z. bailii* IST302 containing the recombinant vector with the extra copy of either *ZbMSN4* or *ZbTIF3* is significantly more tolerant to acetic acid in comparison with the host strain with the empty expression vector (Fig. 6).

## Discussion

In order to get clues on the genes/proteins involved in the high intrinsic tolerance to acetic acid of the *Z. bailii*-derived interspecies hybrid strain ISA1307, we have explored a previously constructed genomic library from this strain, as well as the available information on its full genome sequence which was released and annotated during the progress of this study [14]. Considering the experimental design of the screening carried out to search for genes required for maximal tolerance to acetic acid, it was not expected that the identification of ISA1307 *HAA1* homologue would fail. Indeed, based on the information retrieved from the PEDANT database, two copies of a putative *HAA1*-homologue are present in the genome of this hybrid strain (ZBAI_01494 and ZBAI_05761) sharing, respectively, 50 and 47 % identity with *S. cerevisiae HAA1* nucleotide sequence (pairwise alignment performed at http://www.ebi.ac.uk/Tools/psa/ (European Bioinformatics Institute)). The fact is that when the genomic library used in the screening was constructed [15], it was assumed that the size of ISA1307 genome was similar to the size of *S. cerevisiae* genome (12 Mb). However, following genome sequencing, the genome size of this hybrid strain was found to have approximately 22 Mb [14]. This means that the genomic library tested is poorly representative of the full ISA1307 genome and thus a full functional *HAA1* gene sequence may be missing in the library. Also, we cannot exclude at this time the possibility that ISA1307 Haa1 proteins do not have in this highly acetic acid tolerant strain the important role demonstrated for the *S. cerevisiae* homologue or that the *HAA1* homologous genes in this strain are not functional.

The genes emerging from this study derive at similar proportions from each of the two parental strains of the hybrid strain ISA1307. Remarkably, the differences registered in the two alleles of ISA1307, at the nucleotide level, have apparently no impact in the amino acid sequence of the encoded proteins identified in our study as potential determinants of tolerance to acetic acid. In fact, only six gene pairs in ISA1307 whole genome exhibit nucleotide differences (rate of non-synonymous substitutions (dN) and synonymous substitutions (dS) above 1) with an impact in the amino acid sequence of the encoded proteins [14].

The mechanisms behind yeast adaptation and tolerance to weak acids stress are multifactorial [9]. In other words, yeast tolerance does not rely in a single key cellular process, so it would be expectable that the candidate determinants of acetic acid tolerance emerging from this study are related to different functions in cell. Indeed, the genes identified in our screening as strong candidate determinants of acetic acid tolerance in the highly tolerant strain ISA1307 include genes presumably involved in functions such as cellular transport and transport routes, protein fate, protein synthesis, amino acid metabolism and transcription, as detailed below. The ORFs identified in this study that share poor or no homology with *S. cerevisiae* genes should also be considered as relevant, in particular ZBAI_09903 that holds a pyrimidine binding domain of pyruvate decarboxylase required for the conversion of pyruvate into acetaldehyde and ethanol. Interestingly, a pyruvate decarboxylase enzyme, involved in pyruvate fermentation to acetaldehyde and ethanol, was recently reported as being up-regulated in acetic acid-grown ISA1307 cells compared to cells grown on glucose [16]. Moreover, the deletion of *PDC1*, coding for *S. cerevisiae* pyruvate decarboxylase, was found to lead to a susceptibility phenotype in cells exposed to acetic and propionic acids [7, 8].

Among the strong candidate determinants of acetic acid tolerance we identified *GYP8* and *WSC4* homologues, putatively involved in cellular transport, in particular vesicular transport. The processes of transport vesicle formation, targeting, docking and tethering require complex molecular machinery. Different Ras-like GTPases contribute to these activities by acting as key regulators in assembling specific protein complexes at different donor and target membranes [21–23]. In *S. cerevisiae*, *GYP8* encodes a GTPase-activating protein for Ypt/Rab GTPases, which plays an essential function in endoplasmic reticulum to Golgi vesicular protein transport. *S. cerevisiae* g*yp8*Δ was found to be sensitive to acetic acid and the expression of the *Z. bailii GYP8* homologue, sharing 99 % identity with *Z. bailii* CLIB213^T^, led to a suppression of the mutant phenotype providing evidences of a similar function in *Z. bailii*. To our best knowledge, this is the first time that this gene is related with increased tolerance to acetic acid in yeast. Also in the context of cellular transport, we found that the expression of *WSC4* homologue (presumably derived from the non-*Z. bailii* parental strain) suppresses the susceptibility phenotype of *S. cerevisiae wsc4*Δ mutant. *S. cerevisiae* Wsc4 is specifically involved in the translocation of soluble secretory proteins and insertion of membrane proteins into the ER membrane. In addition to its role in protein trafficking activity, Wsc4 was also described to play a role in *S. cerevisiae* response to a number of stresses, namely to heat shock, exposure to ethanol or 4-nitroquinoline 1-oxide (4-NQO), a DNA-damaging agent [24]. To our knowledge, there is no published information about the increased susceptibility to weak acids of *wsc4*Δ mutant, but the *WSC4* gene was found to be up-regulated after exposure to acetic acid by two-fold in *S. cerevisiae* [6]. Moreover, it is known that this gene is co-regulated by the weak acid-responsive transcription factors Haa1 and Rim101 in response to acetic acid stress [6].

Genes homologous to *S. cerevisiae RKR1*, *PMT1* and *KTR7*, putatively involved in modification and degradation of proteins, were also considered strong candidate determinants of acetic acid tolerance. In *S. cerevisiae*, both *PMT1* and *KTR7* code for mannosyltransferases that have been described as key enzymes for protein glycosylation known to be essential for cell wall rigidity [25, 26]. The remodelling of cell wall is considered a common response to several environmental stresses [8]. In the specific case of weak acid stress, such remodelling is known to be an essential adaptive response that presumably leads to the reduction of the diffusion rate of the undissociated weak acid form into the cell interior, limiting the futile cycle resulting from the continuous uptake of the lipophilic form by passive diffusion followed by the active expulsion of the counterion through specific transporters [27, 28]. Remarkably, the family-related *S. cerevisiae* gene *KTR4*, encoding another mannosyltransferase involved in protein glycosylation, was previously identified as a determinant of acetic acid tolerance [8].

The *ILV3* homologue was also identified as a strong candidate determinant of acetic acid tolerance. In *S. cerevisiae*, *ILV3* codes for a dihydroxyacid dehydratase that catalyzes the third step in the pathways leading to biosynthesis of branched-chain amino acids [29]. Interestingly, the content of Ilv3 was found to be higher in acetic acid-challenged yeast cells, compared with unstressed cells [30].

Among the DNA inserts identified during the present work, the best inserts whose expression confers a high protective effect against acetic acid stress in both the parental and the deletion mutant strains are S06 and those containing *MSN4* or *TIF3* homologues.

The ORF ZBAI_02295, one of two ORFs included in S06 insert and presumably involved in membrane trafficking, was identified as being responsible for the considerable increase in the acetic acid tolerance of the *haa1*Δ mutant, but not of the parental strain, thereby suggesting that the expression of ZBAI_02295 can support the function of Haa1 targets in *S. cerevisiae* but cannot, at least individually, further enhance the acetic acid tolerance of the parental strain. The contribution of the encoding protein, containing a functional domain associated to the uncharacterised DUP family to acetic acid tolerance, is still unclear. DUP proteins were previously suggested as being connected to membrane trafficking and hypothesized as being involved in the trafficking of membrane transporters [20, 31]. Results gathered from large-scale chemical genomics screenings suggest that intracellular trafficking processes are required for weak acid tolerance [9]. Moreover, the genes of the DUP240 family in *S. cerevisiae YAR028W* and *YAR029W* were found to be up-regulated, their transcription being Haa1 dependent, in cells exposed to acetic acid stress [6], reinforcing the idea that this family of proteins is involved in yeast response and tolerance to acetic acid and that the expression of ZBAI_02295 in *haa1*Δ can compensate the abolishment of *YAR028W* and *YAR029W* transcription and therefore elevate the acetic acid susceptibility of the mutant up to the level of the parental strain. This increase of tolerance was not observed in the parental cells maybe because the putative membrane protein originated from the ORF ZBAI_02295 does not bring any advantage in cells where the Haa1 regulon is transcribed. The fact that the expression of S06 insert, which contains ZBAI_02295 and ZBAI_02296, leads to the increase of the acetic acid tolerance of the parental strain may be due to the conjugated effect of both ORFs and also to the different conditions used, namely the medium composition and the cloning vector. The expression of the genes from S06 DNA insert under the control of their native promoters compared with the individual expression of each gene under the *GAL1* inducible promoter can be different and it is likely that the overproduction of the two putative membrane proteins encoded by these ORFs might be deleterious to the host cell.

The relevance of *TIF3* homologue as a putative translation initiation factor in yeast tolerance to acetic acid may rely on the fact that stressed cells generate stress-responsive programmes consisting on a specific up-regulation of translation initiation towards the induction of specific adaptation proteins, while a rapid inhibition of protein synthesis in general occurs, in order to rationalize the consumption of resources [32]. Using the non-flocculating *Z. bailii* strain IST302, it was possible to confirm that the increased expression of *ZbTIF3* in this same strain enhances its tolerance to acetic acid.

ISA1307 *MSN4* gene is homologous to *S. cerevisiae* paralogous genes encoding the transcription factors Msn2 and Msn4. These transcription factors are involved in the regulation of genes of the general stress response and, despite their high homology, both proteins play non-redundant and condition-specific roles in gene expression regulation in *S. cerevisiae* [33]. Interestingly, it was found that both the Haa1 and Msn2/4 regulons share a total of 23 acetic acid-activated genes [6]. For this reason, it is likely that the strain ISA1307 Msn4 homologue has the ability to suppress *haa1*Δ susceptibility phenotype in *S. cerevisiae*. Remarkably, ISA1307 Msn4 homologue was indeed one of the best acetic acid susceptibility suppressors found in the screening. Moreover, the expression of *ZbMSN4* from IST302 either in *S. cerevisiae* parental strains BY4741 and W303-1A or the derived deletion mutants *msn4*Δ and *msn2*Δ*msn4*Δ confers increased protection against acetic acid, indicating that ZbMsn4 has a role similar to its homologous *S. cerevisiae* protein. In addition, the increased expression of *ZbMSN4* in *Z. bailii* IST302 also elevated this strain’s tolerance to acetic acid, confirming this putative transcription factor as an important determinant of acetic acid resistance in *Z. bailii*.

## Conclusions

In the present study we have identified several strong candidate determinants of tolerance to acetic acid in the intrinsically highly acetic acid tolerant *Z. bailii*-derived hybrid strain ISA1307. Specifically, this is the case of the following *S. cerevisiae* homologues presumably involved in different cellular processes - *GYP8* and *WSC4* (cellular transport and transport routes), *PMT1*, *KTR7* and *RKR1* (protein fate), *TIF3* (protein synthesis), *ILV3* (amino acid metabolism), *MSN4* (transcription) - and of ZBAI_02295 (homologous to membrane proteins of unknown function and presumably involved in cellular trafficking). The overexpression of *ZbMSN4* and *ZbTIF3* genes, which were confirmed as playing a relevant role in acetic acid tolerance in yeast through their homologous and heterologous expression in *Z. bailii* and *S. cerevisiae* strains, may be useful considering the improvement of yeast cell robustness against acetic acid if the objective is their use as cell factories.

## Methods

### Strains and growth media

The *Saccharomyces cerevisiae* deletion mutant strain BY4741_*haa1*Δ, derived from the parental strain BY4741 (*MATa, his3*Δ*1, leu2*Δ*0, met15*Δ*0, ura3*Δ*0*) and obtained from the EUROSCARF collection was used as the host strain for the screening of acetic acid determinants of tolerance in a *Zygosaccharomyces bailii*-derived interspecies hybrid strain ISA1307 using a previously constructed genomic library [15]. Strains *gyp8*Δ*, ktr7*Δ*, rkr1*Δ*, msn4*Δ*, pmt1*Δ*, tif3*Δ and *wsc4*Δ derived from the same *S. cerevisiae* parental strain were used for phenotype complementation assays. Since the deletion of *ILV3* gene in the haploid background strain BY4741 is lethal, the *ilv3*Δ mutant used in phenotype complementation assays was derived from the diploid parental strain BY4743 (*MATa/a*, *his3*Δ*1/his3*Δ*1*, *leu2*Δ*0/leu2*Δ*0*, lys2Δ0/*LYS2*, *MET15/met15*Δ*0*, *ura3*Δ*0/ura3*Δ*0*) with one copy of *ILV3* deleted. *S. cerevisiae* strains W303-1A (*MAT*a, *leu2*Δ*3*, *112 trp1*Δ*1*, *can1*Δ*100*, *ura3*Δ*1*, *ade2*Δ*1*, *his3*Δ*11,15*) and derived double deletion mutant *msn2*Δ*msn4*Δ [34] were used for complementation assays of *MSN4* from *Z. bailii* IST302 (*ZbMSN4*). This strain was isolated from a spontaneous fermentation of grape must from Douro wine-producing areas and its genome was recently sequenced and annotated in our laboratory (unpublished results). The taxonomic identification of *Z. bailii* IST302 was confirmed by comparing the partial 26S ribosomal DNA sequence (Additional file 3) with other DNA sequences from *Z. bailii* strains using the Basic Local Alignment Search Tool (BLAST) of the National Center for Biotechnology Information (NCBI). Strain IST302 was used for the amplification of *ZbMSN4* and *ZbTIF3* genes that were cloned into pZ_3_*b*T. This strain was also used as a host cell for the expression of an extra copy of its *ZbMSN4* and *ZbTIF3* genes. *Escherichia coli* XL1-Blue was used for plasmid maintenance and general cloning procedures. *E. coli* cells were grown in Luria-Bertani medium (LB), supplemented with 150 μg/ml ampicillin when required. Yeast strains were batch-cultured at 30 °C with orbital agitation (250 rpm) in liquid mineral medium (MM) that contains, per litre: 1.7 g yeast nitrogen base without amino acids or (NH_4_)_2_SO_4_ (Difco Laboratories, Detroit, Mich.), 20 g glucose (Merck) and 2.65 g (NH_4_)_2_SO_4_ (Merck). Supplementation was needed for auxotrophic strains either with 20 mg methionine, 20 mg histidine, 60 mg leucine and 20 mg uracil for growth of BY4741 and BY4743 background strains (MM4), or with 20 mg histidine, 60 mg leucine, 40 mg tryptophan, 80 mg adenine and 20 mg uracil for growth of W303-1A background strains (MM5) (all from Sigma, Spain). For transformants selection, yeast strains were grown in MM4 or MM5 media without uracil supplementation. YPD medium (2 % glucose (Merck), 1 % yeast extract (Difco) and 2 % peptone (Difco)) was used for yeast cells’ maintenance and to grow cells for transformation experiments. Solid media were obtained by adding 20 g of agar to each litre of the corresponding liquid media. All strains were maintained at –80 °C in appropriate media supplemented with 15 % glycerol (v/v).

### Screening of the genomic library from *Z. bailii-*derived interspecies hybrid strain ISA1307

#### Transformation of *S. cerevisiae* BY4741_*haa1*Δ with strain ISA1307 genomic library

The ISA1307 strain genomic library constructed by Rodrigues et al. [15] in the pRS316 vector [35] was used in this work. This plasmid is a shuttle vector for *S. cerevisiae* and *E. coli*, containing origins of replication for both species and Amp^r^ and *URA3* genes as selection markers. Moreover, this cloning vector is a centromeric plasmid, possessing an *S. cerevisiae* centromere (CEN6). The choice for a low copy plasmid is useful for isolation of genes that may have a toxic effect when present in multiple copies. For library plasmid DNA isolation, *E. coli* cells containing the genomic library were grown at 26 °C on LB medium supplemented with ampicillin until late stationary-phase and plasmid DNA was extracted from these cells using the QIAprep® Spin Maxiprep Kit according to the manufacturer’s instructions. *S. cerevisiae* BY4741*_haa1*Δ strain was transformed with library plasmid DNA, using the lithium acetate method [36]. The transformation mixture was plated onto solid MM4 medium without uracil and plates were incubated at 30 °C for 3 days.

#### Screening of the transformants for acetic acid tolerance

Selection of the transformants showing more tolerance to acetic acid was performed in 96-well microplates containing liquid MM4 medium without uracil at pH 4.0. At the end of the day, a first plate containing 200 μl of liquid medium was inoculated with cells taken from the different yeast transformants under study, and incubated at 30 °C overnight with orbital agitation at 250 rpm. On the next morning, each inoculum was diluted and used to inoculate a microplate containing the same liquid media supplemented with 60 mM of acetic acid, at an initial OD_600nm_ of about 0.05. This plate was incubated at 30 °C for 24 h under stirring at 250 rpm and after that time period the culture OD_600 nm_ was measured (VersaMax microplate reader*,* Molecular Devices). The tolerance of transformants to acetic acid was based on these values of growth observed after 24 h of incubation. Candidates were considered susceptible to acetic acid when OD_600nm_ reached values below 0.250, while candidates with an intermediate tolerance phenotype to acetic acid exhibited an OD_600nm_ between 0.250 and 0.350 and the tolerant candidates an OD_600nm_ above 0.350. *S. cerevisiae* BY4741_*haa1*Δ was used as the negative control as it is particularly susceptible to 60 mM acetic acid, pH 4.0, being unable to grow within the period of incubation used.

### Comparison of the tolerance of the selected candidates to acetic acid

A confirmation screening of acetic acid tolerance phenotype was performed with the isolation of total DNA from yeast transformants selected as the most tolerant to acetic acid. DNA was used to transform *E. coli* XL1-Blue strain competent cells by electroporation using Bio-Rad Gene Pulser II (400 Ω, 25 μF, 2.5 kV). The transformants obtained were selected for ampicillin resistance and cultivated for 12 h in 3 ml of LB liquid medium with ampicillin at 37 °C with an orbital agitation of 250 rpm. For plasmid isolation from *E. coli* a QIAprep® Spin Miniprep Kit was used, according to the manufacturer’s instructions. The extracted DNA was then used to transform the parental strain *S. cerevisiae* BY4741 and the derived deletion mutant BY4741_*haa1*Δ, using the lithium acetate method [36]. Growth curves of these strains in liquid media, either in the absence or presence of acetic acid were determined. Cells were grown in MM4 medium without uracil at pH 4.0 until exponential phase (OD_600nm_ of 0.5 ± 0.05) and then re-inoculated at an OD_600nm_ of 0.05, in 50 ml of fresh medium, either or not supplemented with 60 mM of acetic acid. Growth was followed by measuring culture OD_600nm_ during batch cultivation at 30 °C with an orbital agitation of 250 rpm. The parental strain *S. cerevisiae* BY4741 and the mutant BY4741_*haa1*Δ both transformed with the empty vector were used as controls. The suppressors of acetic acid susceptibility phenotype of BY4741_*haa1*Δ were selected and submitted to further sequence analysis. Finally, an additional confirmation step of the phenotype of BY4741_*haa1*Δ cells transformed with the sequenced suppressors was performed by repeating their growth curves in control conditions and in the presence of 60 mM of acetic acid in 96-well microplates (FilterMax F5, Multi-mode microplate reader, Molecular Devices), at 30 °C. Microplate reads were carried out every 10 min using a 595 nm absorbance filter. An orbital agitation of three seconds was performed prior to each read. The growth curves of *S. cerevisiae* BY4741 parental strain transformed with these same suppressors were repeated as well. The plates were inoculated with cells in exponential phase (OD_600nm_ of 0.5 ± 0.05) at an initial OD_600nm_ of 0.05. In order to be comparable, all the growth curves must come from the same plate, since the level of inhibition of the acid is hardly reproducible from plate to plate due to the extremely small volume contained in each well. So, all the parental strain transformants were grown in the same plate, and the same was done for the transformants of the deletion mutant BY4741_*haa1*Δ. For comparison purposes between the transformants from the two different backgrounds, the growth curve of the parental strain with the empty vector was included in the plates with the BY4741_*haa1*Δ transformants.

### *In silico* analysis of the selected plasmid DNA inserts from strain ISA1307 genomic library

The complete DNA sequence of the selected plasmid inserts from strain ISA1307 genomic library was obtained in two steps. First, approximately 1000 bp of the DNA sequence ends of each plasmid insert were sequenced using vector specific primers (forward and reverse). Second, the complete sequence of the plasmid insert was acquired by alignment of the sequenced ends against the genome sequence of the strain ISA1307 available in the PEDANT database (http://pedant.helmholtz-muenchen.de/genomes.jsp?Category=fungal) [37]. This sequence was part of a high-throughput DNA sequencing project, based on Illumina paired-end sequencing, carried out by our group [14]. The *in silico* analysis of each DNA plasmid insert from the genomic library was performed also using the PEDANT database and the genomic browser GBrowse_syn (http://mips.helmholtz-muenchen.de/gbrowse2/cgi-bin/gbrowse_syn/zbailii) [38] that returns all the putative open reading frames (ORFs) present in the DNA fragment based on sequence homology with *S. cerevisiae*. Additionally, this tool provides the synteny alignments for the *Z. bailii*-derived interspecies hybrid strain ISA1307 genome versus the genomes of *Z. bailii* CLIB213^T^, *Z. rouxii* and *S. cerevisiae*. When the plasmid DNA insert contained truncated ORFs, the presence of specific functional domains was assessed using the Conserved Domain Database of the NCBI [19]. The description of each gene was based on the information gathered in *Saccharomyces* Genome Database (www.yeastgenome.org) for the corresponding putative homologue.

### Cloning and expression of ZBAI_02295 and ZBAI_02296 in *S. cerevisiae*

The pGREG506 plasmid from the DRAG & DROP collection [39] was used to individually clone by homologous recombination and express the genes ZBAI_02295 and ZBAI_02296 identified in the S06 DNA fragment from ISA1307 genomic library. That cloning vector was acquired from Euroscarf and contains a *HIS3* gene under the control of a galactose inducible promoter (*GAL1*) and the yeast selectable marker *URA3*. During homologous recombination the *HIS3* gene is replaced by the gene of interest. ZBAI_02295 and ZBAI_02296 DNA fragments were generated by PCR using genomic DNA extracted from the strain ISA1307 and the specific primers ZBAI_02295_FWD, ZBAI_02295_REV, ZBAI_02296_FWD and ZBAI_02296_REV, whose sequences are listed in Additional file 4. The cDNA from each gene was co-transformed into the parental strain BY4741 and derived deletion mutant *haa1*Δ with the pGREG506 vector, previously cut with *SalI* restriction enzyme. The recombinant plasmids pGREG506_ ZBAI_02295 and pGREG506_ ZBAI_02296 were obtained through homologous recombination in yeast. Correct cloning of each ORF was confirmed by DNA sequencing. Additionally, *S. cerevisiae* BY4741 parental and derived deletion mutant *haa1*Δ strains were transformed with the cloning vector pGREG506. To have the adequate control cells, both strains were transformed as well with the empty vector pGREG506 with the *HIS3* gene deleted (pGREG506_noHIS3, obtained by digestion of pGREG506 with the restriction enzyme *SalI*), since in the recombinant vector the *HIS3* gene is substituted by the insert of interest. The susceptibility to acetic acid of the parental strain BY4741 and the derived deletion mutant *haa1*Δ, harbouring each one of the four described plasmids (pGREG506_ ZBAI_02295, pGREG506_ ZBAI_02296, pGREG506 and pGREG506_noHIS3) was assessed. This was possible by comparing their growth curves in liquid MM4-U medium supplemented with 1 % of galactose (w/v) pH 4.0, at 30 °C with orbital agitation (250 rpm), either or not supplemented with 60 mM acetic acid, as previously described.

### Complementation of *S. cerevisiae* deletion mutants by the corresponding *Z. bailii* ISA1307 single genes present in genomic library plasmids

The genomic library plasmids 8.23, 18.22, 23.1, B02, B18, S07 and Y08 carrying the single *S. cerevisiae* putative homologous genes *GYP8*, *PMT1*, *WSC4*, *MSN4*, *TIF3*, *KTR7* or *RKR1*, respectively, were transformed in *S. cerevisiae* BY4741 EUROSCARF mutants lacking the respective gene. The complementation of library plasmid 23.17 carrying *ILV3* was performed using the diploid BY4743 parental strain with one copy of the essential *ILV3* gene deleted, because the haploid BY4741_*ilv3*Δ is unviable. Both *S. cerevisiae* parental strains transformed with the empty vector were used as control. The transformants were batch-cultivated in MM4-U medium at pH 4.0, either or not supplemented with 60 mM of acetic acid, as previously described. Since *S. cerevisiae tif3*Δ is highly susceptible to acetic acid, a growth medium containing a lower concentration of acetic acid (40 mM) was used. The complementation assays using the diploid strains derived from BY4743 were performed with a higher concentration (65 mM) of acetic acid.

### Cloning and expression of *ZbMSN4* in *S. cerevisiae*

The gene *ZbMSN4* amplified from *Z. bailii* IST302 was cloned by homologous recombination into pGREG506 using the strategy above described for the cloning of ZBAI_02295 and ZBAI_02296. The primers ZbMSN4_pGREG_FWD and ZbMSN4_pGREG_REV (Additional file 4) were used for the amplification of *ZbMSN4* coding region. This DNA fragment and the pGREG506 vector previously digested with *SalI* restriction enzyme were co-transformed into *S. cerevisiae* BY4741 parental strain. The obtained recombinant plasmid pGREG506_ZbMSN4 was digested with *Spe*I and *Asc*I restriction enzymes to remove the *GAL1* promoter from the vector. The digested vector and *S. cerevisiae MSN4* promoter (approximately 1000 bp upstream the start codon) that was amplified with primers ZbMSN4prom_FWD and ZbMSN4prom_REV (Additional file 4) were co-transformed into *S. cerevisiae* BY4741 parental cells. The recombinant vector pGREG506_promMSN4Sc_ZbMSN4 obtained by homologous recombination was sequenced to confirm the correct cloning of the promoter region and the gene and transformed afterwards into *S. cerevisiae* BY4741_*msn4*Δ, W303-1A parental and derived double deletion mutant *msn2*∆*msn4*∆ strains. The susceptibility to acetic acid of the parental strains BY4741 and W303-1A and of the corresponding derived deletion mutants *msn4*Δ and *msn2*∆*msn4*Δ, harbouring pGREG506_promMSN4Sc_ZbMSN4 and also pGREG506_noHIS3 was assessed by comparing their growth curves in liquid MM4-U (BY4741 background strains) and MM5-U (W303-1A background strains), either or not supplemented with the appropriate acetic acid concentration.

### Cloning and expression of an extra copy of *ZbMSN4* and *ZbTIF3* in *Z. bailii* IST302

The expression of an extra copy of *ZbMSN4* and *ZbTIF3* genes in the parental strain *Z. bailii* IST302 was performed by cloning these genes by homologous recombination into the centromeric expression vector pZ_3_*b*T [40] linearized with *XbaI*. The genes *ZbMSN4* and *ZbTIF3* and their corresponding promoters (approximately 1000 bp upstream the start codon) were amplified from strain IST302 genomic DNA using the primers ZbMSN4_FWD, ZbMSN4_REV, ZbTIF3_FWD and ZbTIF3_REV (Additional file 4) and co-transformed with the linearized pZ_3_*b*T into *S. cerevisiae* BY4741. Selection of the transformants holding the recombinant vectors pZ_3_*b*T_ *ZbMSN4* and pZ_3_*b*T_ *ZbTIF3* was performed in YPD plates containing G418 (200 mg/L). The correct recombination was confirmed by sequencing the obtained plasmids. The recombinant plasmids and the empty plasmid were used to transform *Z. bailii* IST302 using a commercial yeast transformation kit (MP Biomedicals, California) with minor modifications. The susceptibility to acetic acid of *Z. bailii* IST302 cells transformed with pZ_3_*b*T or with the recombinant vectors pZ_3_*b*T_ *ZbMSN4* and pZ_3_*b*T_ *ZbTIF3* was assessed by comparing their growth curves in liquid MM either or not supplemented with 180 or 220 mM acetic acid.

## Availability of supporting data

All the supporting data of this article is included within the article and in its additional files. The partial sequence of the 26S ribosomal RNA gene of *Z. bailii* strain IST302 (Additional file 3) is deposited in GeneBank (accession number: KU194200).

## Additional files

Additional file 1: Figure S1. Growth curves of the 31 transformants selected as the best candidates. *S. cerevisiae* BY4741_*haa1*Δ (A) and BY4741 parental (B) strains transformed with the empty vector (ø) and with the 31 selected vectors from ISA1307 genomic library (legend included in the chart). Yeast cells were grown in MM4 medium (pH 4.0) without uracil, supplemented with 60 mM acetic acid. The curves are representative of at least three independent assays. (PPTX 675 kb) Additional file 2:
**Genomic DNA inserts from the strain ISA1307 found to rescue the acetic acid susceptibility phenotype of** ***S. cerevisiae*** **BY4741_** ***haa1Δ.*** Based on ISA1307 annotated genome, several complete and incomplete ORFs were identified in each fragment. (PDF 182 kb) Additional file 3:
**Partial sequence of the 26S ribosomal RNA gene of** ***Z. bailii*** **strain IST302.** (PDF 4 kb) Additional file 4:
**List of primers used in this work.** All the primers contain a region with homology to the gene/region to be amplified (italics) and a nucleotide sequence with homology to the cloning site flanking region of the vector used for recombination (underlined). (PDF 170 kb)
