# Supplementary material for: Search for genes responsible for the remarkably high acetic acid tolerance of a Zygosaccharomyces bailii-derived interspecies hybrid strain
Source: BMC Genomics. 2015 Dec 16;16:1070. doi: 10.1186/s12864-015-2278-6 (PMC4681151; doi:10.1186/s12864-015-2278-6)
Supplement: Additional file 1: Figure S1. — Growth curves of the 31 transformants selected as the best candidates. S. cerevisiae BY4741_haa1Δ (A) and BY4741 parental (B) strains transformed with the empty vector (ø) and with the 31 selected vectors from ISA1307 genomic library (legend included in the chart). Yeast cells were grown in MM4 medium (pH 4.0) without uracil, supplemented with 60 mM acetic acid. The curves are representative of at least three independent assays. (PPTX 675 kb) [file 12864_2015_2278_MOESM1_ESM.pptx]

## Slide 1
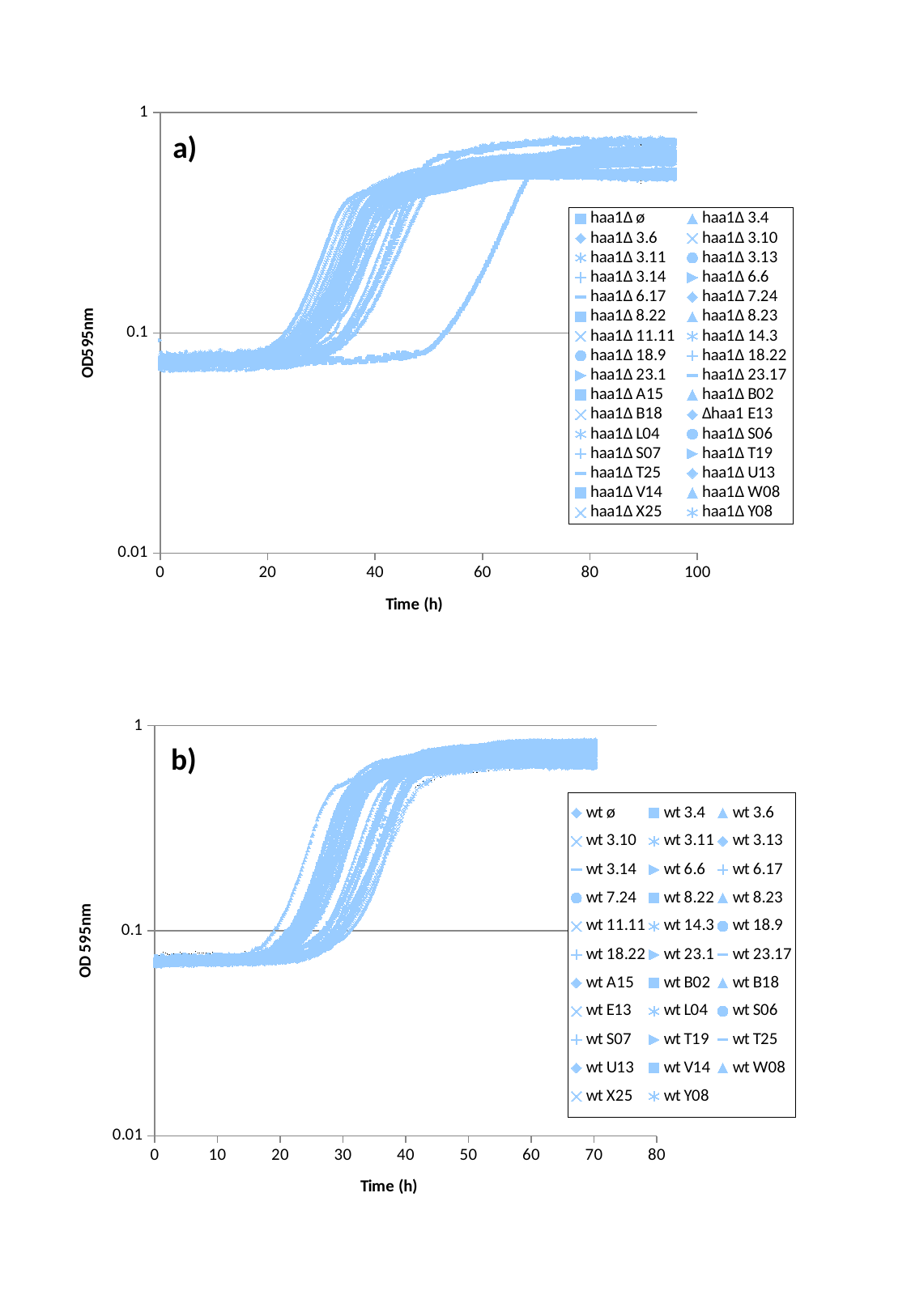

### Chart
| Category | haa1∆ ø | haa1∆ 3.4 | haa1∆ 3.6 | haa1∆ 3.10 | haa1∆ 3.11 | haa1∆ 3.13 | haa1∆ 3.14 | haa1∆ 6.6 | haa1∆ 6.17 | haa1∆ 7.24 | haa1∆ 8.22 | haa1∆ 8.23 | haa1∆ 11.11 | haa1∆ 14.3 | haa1∆ 18.9 | haa1∆ 18.22 | haa1∆ 23.1 | haa1∆ 23.17 | haa1∆ A15 | haa1∆ B02 | haa1∆ B18 | ∆haa1 E13 | haa1∆ L04 | haa1∆ S06 | haa1∆ S07 | haa1∆ T19 | haa1∆ T25 | haa1∆ U13 | haa1∆ V14 | haa1∆ W08 | haa1∆ X25 | haa1∆ Y08 |
|---|---|---|---|---|---|---|---|---|---|---|---|---|---|---|---|---|---|---|---|---|---|---|---|---|---|---|---|---|---|---|---|---|a)
### Chart
| Category | wt ø | wt 3.4 | wt 3.6 | wt 3.10 | wt 3.11 | wt 3.13 | wt 3.14 | wt 6.6 | wt 6.17 | wt 7.24 | wt 8.22 | wt 8.23 | wt 11.11 | wt 14.3 | wt 18.9 | wt 18.22 | wt 23.1 | wt 23.17 | wt A15 | wt B02 | wt B18 | wt E13 | wt L04 | wt S06 | wt S07 | wt T19 | wt T25 | wt U13 | wt V14 | wt W08 | wt X25 | wt Y08 |
|---|---|---|---|---|---|---|---|---|---|---|---|---|---|---|---|---|---|---|---|---|---|---|---|---|---|---|---|---|---|---|---|---|b)
