# Supplementary material for: Search for genes responsible for the remarkably high acetic acid tolerance of a Zygosaccharomyces bailii-derived interspecies hybrid strain
Source: BMC Genomics. 2015 Dec 16;16:1070. doi: 10.1186/s12864-015-2278-6 (PMC4681151; doi:10.1186/s12864-015-2278-6)
Supplement: Additional file 2: — Genomic DNA inserts from the strain ISA1307 found to rescue the acetic acid susceptibility phenotype of S. cerevisiae BY4741_ haa1Δ. Based on ISA1307 annotated genome, several complete and incomplete ORFs were identified in each fragment. (PDF 182 kb) [file 12864_2015_2278_MOESM2_ESM.pdf]

| DNA insert | ORF [(a)] | Identity (%) with *Z. bailii* CLIB213$^{T}$ [(b)] | *S.cerevisiae* putative homologue | Identity (%) with *S. cerevisiae* [(c)] | Size (aa) ISA1307 / *S. cerevisiae* / Alignment [(d)] | *S.cerevisiae* function [(e)] | Alteration in yeast deletion mutant susceptibility to weak organic acids [(f)] |
|---|---|---|---|---|---|---|---|
| **3.4** (3205 bp) | ZBAI_04394 | 98 | *RPL27A/RPL27B* | 91.1 | 80 / 136 / 79 | Ribosomal 60S subunit protein L27A/L27B **Protein synthesis** | **Acetic acid** (S) [1] |
| | ZBAI_04395 (73%) | 93/93 | *DIA4* | 58.0 | 444 / 446 / 445 | Probable mitochondrial seryl-tRNA synthetase **Protein synthesis** | - |
| **3.6** (4247 bp) | ZBAI_09932 | 97 | *SAR1* | 93.0 | 189/191/173 | GTPase required for transport vesicle formation during ER to Golgi protein transport **Cellular transport and transport routes** | - |
| | ZBAI_04613 | 93 | *PCL10* | 36.6 | 451 / 433 / 435 | Cyclin involved in regulation of activity of Pho85p protein kinase **C-compound and carbohydrate metabolism** | **Acetic acid** (S) [1] |
| | ZBAI_04614 | 97 | *RPL1A/RPL1B* | 89.4 | 217 / 217 / 217 | Ribosomal 60S subunit protein L1A/L1B **Protein synthesis** | **Acetic acid** (S) [1,2] |
| **3.10** (4903 bp) | ZBAI_02850 | 95 | *YDR056C* | 37.6 | 200 / 205 / 194 | Putative protein of unknown function **Unclassified** | - |
| | ZBAI_02851 | 94 | *YOS9* | 33.0 | 547 / 542 / 525 | Endoplasmic reticulum quality-control lectin **Cellular transport and transport routes** | **Acetic acid** (S) [1] |
| | ZBAI_02852 (57%) | 93/94 | *SPT7* | 54.0 | 1261 / 1332 / 1342 | Subunit of the SAGA transcriptional regulatory complex, also present as a C-terminally truncated form in the SLIK/SALSA transcriptional regulatory complex **Transcription** | **Mycophenolic acid** (S) [3] |
| **3.11** (4601 bp) | ZBAI_04683 (27%) | 96/93 | *KIP1* | 41.5 | 1134 / 1111 / 1156 | Kinesin-related motor protein required for mitotic spindle assembly, chromosome segregation, and 2 micron plasmid partitioning. **Cell cycle and DNA processing** | - |
| | ZBAI_04684 | 96 | *RHO1* | 79.3 | 215 / 209 / 203 | GTPase involved in establishment of cell polarity **Cytoskeleton/structural proteins** | - |
| | ZBAI_04685 | 96 | *MRP2* | 73.0 | 115 / 115 / 115 | Mitochondrial ribosomal 37S subunit protein **Protein synthesis** | - |

| | | | | | | | |
|---|---|---|---|---|---|---|---|
| **3.13** (4069 bp) | ZBAI_06218 (47%) | 99/99 | *TUP1* | 64.9 | 678 / 713 / 724 | General repressor of transcription<br>**Transcription** | **Acetic acid** (S) [8] |
| | ZBAI_06219 | 100 | *CSM1* | 50.3 | 177 / 190 / 191 | Nucleolar protein that mediates accurate homolog segregation during meiosis<br>**Cell cycle and DNA processing** | - |
| | ZBAI_06220 | 100 | *YCR087C-A* | 54.5 | 195 / 153 / 145 | Putative protein of unknown function<br>**Unclassified** | - |
| | ZBAI_06221 (40%) | 100/100 | - | - | 278 / - / - | - | - |
| **3.14** (3027 bp) | ZBAI_05501 | 99 | *END3* | 57.8 | 366 / 349 / 367 | EH domain-containing protein involved in endocytosis, actin cytoskeletal organization and cell wall morphogenesis<br>**Cellular transport and transport routes** | **Acetic acid** (S) [1]<br>**Propionic acid** (S) [4]<br>**Sorbic acid** (S) [5] |
| | ZBAI_05502 (29%) | 99/99 | *CCT2* | 88.4 | 527 / 527 / 527 | Subunit beta of the cytosolic chaperonin Cct ring complex required for the assembly of actin and tubulins in vivo<br>**Protein fate** | - |
| **6.6** (3567 bp) | ZBAI_05952 | 98 | *DML1* | 45.7 | 455 / 475 / 475 | Essential protein involved in mtDNA inheritance, may also function in the partitioning of the mitochondrial organelle or in the segregation of chromosomes.<br>**Cell cycle and DNA processing** | - |
| | ZBAI_05953 (50%) | 100/100 | *MOD5* | 62.0 | 418 / 428 / 413 | Delta 2-isopentenyl pyrophosphate:tRNA isopentenyl transferase<br>**Transcription** | - |
| **6.17** (4408 bp) | ZBAI_01425 (23%) | 96/96 | *ERG3* | 66.2 | 366 / 365 / 367 | C-5 sterol desaturase, glycoprotein that catalyzes the introduction of a C-5(6) double bond into episterol, a precursor in ergosterol biosynthesis<br>**Lipid, fatty acid and isoprenoid metabolism** | **Acetic acid** (S) [1,2]<br>**Lactic acid** (S) [2]<br>**Propionic acid** (S) [4]<br>**Sorbic acid** (S) [5,6]<br>**Mycophenolic acid** (S) [3] |
| | ZBAI_01426 | 94 | *CAF16* | 66.5 | 276 / 289 / 275 | Part of evolutionarily-conserved CCR4-NOT regulatory complex<br>**Transcription** | - |
| **7.24** (5392 bp) | ZBAI_08540 (48%) | 99/99 | *ERB1* | 76.7 | 801 / 807 / 803 | Constituent of 66S pre-ribosomal particles required for maturation of the 25S and 5.8S ribosomal RNAs<br>**Transcription** | - |
| | ZBAI_08541 | 99 | *HIM1* | 36.9 | 417 / 414 /420 | Protein of unknown function involved in DNA repair<br>**Cell cycle and DNA processing** | - |
| | ZBAI_08542 (49%) | 100/99 | *PCM1* | 53.0 | 489 / 557 /504 | Essential N-acetylglucosamine-phosphate mutase<br>**C-compound and carbohydrate metabolism** | - |

| | | | | | | | |
|---|---|---|---|---|---|---|---|
| **8.22** (6332 bp) | ZBAI_08004 (79%) | 99/99 | *OSH2* | 43.9 | 1683 / 1283 / 1657 | Lipid-binding protein involved in maintenance of intracellular sterol distribution and homeostasis **Lipid, fatty acid and isoprenoid metabolism** | - |
| | ZBAI_08005 | 100 | *THI20* | 63.0 | 550 / 551 /546 | Trifunctional enzyme with hydroxymethylpyrimidine (HMP) kinase, HMP-phosphate (HMP-P) kinase and thiaminase activities, involved in thiamine biosynthesis and degradation **Metabolism of vitamins, cofactors, and prosthetic groups** | - |
| **8.23** (2782 bp) | ZBAI_05695 | 99 | ***GYP8*** | 34.0 | 556 / 497 / 503 | GTPase-activating protein involved in the regulation of ER to Golgi vesicle transport **Cellular transport and transport routes** | - |
| **11.11** (5598 bp) | ZBAI_09408 | 100 | *TRM9* | 62.0 | 268 / 279 / 274 | tRNA methyltransferase **Protein synthesis** | - |
| | ZBAI_09409 | 99 | *UBX2* | 38.3 | 611 / 584 / 614 | Protein involved in ER-associated protein degradation **Protein fate** | **Acetic acid** (R) [2] **Lactic acid** (R) [2] |
| | ZBAI_09410 | 100 | *NPL3* | 65.9 | 409 / 414 / 402 | RNA-binding protein that promotes elongation, regulates termination, and carries poly(A) mRNA from nucleus to cytoplasm; required for pre-mRNA splicing. **Cellular transport and transport routes** | **Acetic acid** (S) [1, 2] |
| **14.3** (5139 bp) | ZBAI_08065 (21%) | 99/99 | *SRP101* | 64.1 | 602 / 621 / 622 | Signal recognition particle (SRP) receptor alpha subunit involved in SRP-dependent protein targeting **Cellular transport and transport routes** | - |
| | ZBAI_08066 | 99 | *OLE1* | 35.0 | 534 / 510 / 389 | Delta-9 fatty acid desaturase, required for monounsaturated fatty acid synthesis **Lipid, fatty acid and isoprenoid metabolism** | - |
| | ZBAI_08067 | 99 | *BCD1* | 54.6 | 351 / 366 / 366 | Essential protein required for box C/D snoRNAs accumulation involved in snoRNA processing, snoRNA transport to the nucleolus and ribosome biogenesis **Transcription** | - |
| **18.9** (4511 bp) | ZBAI_03401 (28%) | 95/93 | *YCR016W* | 39.8 | 321 / 290 / 329 | Putative protein of unknown function **Unclassified** | - |
| | ZBAI_03402 | 99 | *YCR015C* | 43.2 | 305 / 317 / 317 | Putative protein of unknown function **Unclassified** | - |
| | ZBAI_03403 | 100 | - | - | 316 / - / - | - | - |
| | ZBAI_03404 (30%) | 99/99 | *POL4* | 38.7 | 571 / 582 / 589 | DNA polymerase IV **Cell cycle and DNA processing** | - |

| | | | | | | | |
|---|---|---|---|---|---|---|---|
| **18.22** (2803 bp) | ZBAI_04770 (41%) | 95/95 | ***PMT1*** | 66.9 | 761 / 817 / 746 | Protein O-mannosyltransferase **Protein fate** | **Acetic acid** (S) [1] |
| **23.1** (2277 bp) | ZBAI_09707 (40%) | 92/95 | ***WSC4*** | 29.1 | 854 / 605 / 632 | ER membrane protein involved in the translocation of soluble secretory proteins and insertion of membrane proteins into the ER membrane; may also have a role in the stress response **Cellular transport and transport routes** | - |
| **23.17** (1812 bp) | ZBAI_09663 (66%) | 99/99 | ***ILV3*** | 82.5 | 583 / 585 / 584 | Dihydroxyacid dehydratase, catalyzes third step in the common pathway leading to biosynthesis of branched-chain amino acids **Amino acid metabolism** | - |
| **A15** (4101+5478 = 9579 bp) | ZBAI_09122 (66%) | 95/95 | *RIB1* | 78.2 | 305 / 345 / 293 | GTP cyclohydrolase II which catalyzes the first step of the riboflavin biosynthesis pathway **Metabolism of vitamins, cofactors, and prosthetic groups** | - |
| | ZBAI_09123 (46%) | 94/94 | *STU1* | 35.4 | 1462 / 1513 / 1550 | Encodes an essential component of the yeast mitotic spindle **Cytoskeleton/structural proteins** | - |
| **B02** (3640 bp) | ZBAI_03527 (77%) | 95/95 | ***MSN4*** | 32.9 | 574 / 630 / 590 | Transcriptional activator that regulates the general stress response of *S. cerevisiae* **Transcription** | **2,4-D** (S) [7] |
| **B18** (2917 bp) | ZBAI_01028 | 94 | ***TIF3*** | 55.8 | 433 / 436 / 453 | Translation initiation factor eIF-4B **Protein synthesis** | **Acetic acid** (S) [2] **Propionic acid** (S) [4] |
| **E13** (4470 bp) | ZBAI_09903 | 95 | - | - | 140 / - / - | - | - |
| | ZBAI_09904 | - | - | - | 117 / - / - | - | - |
| **L04** (3017 bp) | ZBAI_08981 (15%) | 100/99 | *TSR1* | 63.6 | 798 / 788 / 797 | Protein required for processing of 20S pre-rRNA in the cytoplasm **Transcription** | - |
| | ZBAI_08982 | 99 | *REH1* | 53.0 | 410 / 432 / 434 | Cytoplasmic 60S subunit biogenesis factor **Protein fate** | - |
| | ZBAI_08983 (51%) | 100/99 | *VAC14* | 66.5 | 813 / 880 / 824 | Protein involved in regulated synthesis of Phosphatidylinositol 3,5-bisphosphate, in control of trafficking of some proteins to the vacuole lumen via the MVB, and in maintenance of vacuole size and acidity **Lipid, fatty acid and isoprenoid metabolism** | - |

| | | | | | | | |
|---|---|---|---|---|---|---|---|
| **S06**<br>(4413 bp) | **ZBAI_02295** | 99 | - | - | 178 / - / - | - | - |
| | ZBAI_02296 | 98 | - | - | 261 / - / - | - | - |
| **S07**<br>(2860 bp) | ZBAI_05420 (57%) | 95/95 | ***KTR7*** | 49.3 | 514 / 517 / 525 | Putative mannosyltransferase involved in protein glycosylation<br>**Protein fate** | - |
| **T19**<br>(4257 bp) | ZBAI_04654 | 95 | *YPL247C* | 53.7 | 507 / 523 / 547 | Putative protein of unknown function<br>**Unclassified** | - |
| | ZBAI_04655 (69%) | 93/94 | *GCV2* | 70.5 | 1021 / 1034 / 1036 | P subunit of the mitochondrial glycine decarboxylase complex<br>**Amino acid metabolism** | - |
| **T25**<br>(3241 bp) | ZBAI_06968 (87%) | 95/95 | *RAD1* | 58.4 | 1038 / 1100 / 1081 | Single-stranded DNA endonuclease<br>**Cell cycle and DNA processing** | - |
| | ZBAI_06969 (17%) | 95/95 | *SWI1* | 46.3 | 864 / 1314 / 928 | Subunit of the SWI/SNF chromatin remodeling complex<br>**Cell cycle and DNA processing** | - |
| **U13**<br>(2233 bp) | ZBAI_03959 (87%) | 94/94 | *PRO1* | 77.7 | 416 / 428 / 421 | Gamma-glutamyl kinase, catalyzes the first step in proline biosynthesis<br>**Amino acid metabolism** | **Sorbic acid** (R) [6] |
| | ZBAI_03960 (21%) | 92/94 | *CFT1* | 63.7 | 1339 / 1357 / 1357 | RNA-binding subunit of the mRNA cleavage and polyadenylation factor<br>**Transcription** | - |
| **V14**<br>(2907 bp) | ZBAI_09508 | 100 | - | - | 445 / - / - | - | - |
| | ZBAI_09509 (99%) | 100/100 | *TVP18* | 60.0 | 164 / 167 / 165 | Integral membrane protein of unknown function localized to late Golgi vesicles<br>**Unclassified** | **Acetic acid** (S) [1] |
| **W08**<br>(1178 bp) | ZBAI_05704 (81%) | 99/99 | *IES3* | 44.5 | 250 / 250 / 256 | Subunit of the INO80 chromatin remodeling complex<br>**Cell cycle and DNA processing** | **Acetic acid** (S) [1] |
| | ZBAI_05705 (60%) | 100/100 | *FCF2* | 41.9 | 212 / 217 / 215 | Essential nucleolar protein involved in the early steps of 35S rRNA processing<br>**Transcription** | - |

| | | | | | | | |
|---|---|---|---|---|---|---|---|
| **X25** (3949 bp) | ZBAI_07897 | 99 | *AIP1* | 64.6 | 608 / 615 / 615 | Actin cortical patch component involved in the depolymerization of actin filaments **Cytoskeleton/structural proteins** | - |
| | ZBAI_07898 (81%) | 100/100 | *NPL6* | 57.5 | 447 / 435 / 435 | Component of the RSC chromatin remodeling complex involved in nuclear protein import **Cellular transport and transport routes** | **Propionic acid** (S) [4] **Sorbic acid** (S) [6] |
| **Y08** (2104 bp) | ZBAI_01926 (34%) | 92/93 | ***RKR1*** | 48.8 | 1555 / 1562 / 1571 | RING domain E3 ubiquitin ligase; involved in the ubiquitin-mediated degradation of non-stop proteins **Protein fate** | - |

[a] Percentage of nucleotides present in the truncated ORFs, compared with the total ORF sequence, is indicated in parentheses.

[b] Identity (%) between each ORF found in the DNA inserts and *Z. bailii* CLIB213$^T$ genome was obtained using BLAST analysis (http://blast.ncbi.nlm.nih.gov/Blast.cgi).

[c] Identity (%) between ISA1307 and *S. cerevisiae* S288C homologous genes was retrieved from PEDANT database (http://pedant.helmholtz-muenchen.de/genomes.jsp?Category=fungal).

[d] Identity (%) between homologous proteins from ISA1307 and *S. cerevisiae*. The size of the proteins and their pairwise alignment, given by the number of amino acid residues (aa), was retrieved from the PEDANT database (http://pedant.helmholtz-muenchen.de/genomes.jsp?Category=fungal).

[e] The putative function of each ORF was assigned based on the function of each *S. cerevisiae* homologous gene (www.yeastgenome.org). The functional category is provided in bold.

[f] List of the studies involving the *S. cerevisiae* corresponding deletion mutant susceptibility or resistance phenotypes under weak acid stress (S) - the single deletion mutant is susceptible to the acid; (R) - the single deletion mutant is resistant to the acid.

## References

[1] Mira NP, Palma M, Guerreiro JF, Sá-Correia I: **Genome-wide identification of *Saccharomyces cerevisiae* genes required for tolerance to acetic acid.** *Microb Cell Fact* 2010, **9**.

[2] Kawahata M, Masaki K, Fujii T, Iefuji H: **Yeast genes involved in response to lactic acid and acetic acid: acidic conditions caused by the organic acids in *Saccharomyces cerevisiae* cultures induce expression of intracellular metal metabolism genes regulated by Aft1p.** *FEMS Yeast Res* 2006, **6:**924-936.
[3] Desmoucelles C, Pinson B, Saint-Marc C, Daignan-Fornier B: **Screening the yeast "disruptome" for mutants affecting resistance to the immunosuppressive drug, mycophenolic acid.** *J Biol Chem* 2002, **277:**27036-27044.
[4] Mira NP, Lourenço AB, Fernandes AR, Becker JD, Sá-Correia I: **The *RIM101* pathway has a role in *Saccharomyces cerevisiae* adaptive response and resistance to propionic acid and other weak acids.** *FEMS Yeast Res* 2009, **9:**202-216.
[5]Schüller C, Mamnun YM, Mollapour M, Krapf G, Schuster M, Bauer BE, Piper PW, Kuchler K: **Global phenotypic analysis and transcriptional profiling defines the weak acid stress response regulon in *Saccharomyces cerevisiae*.** *Mol Biol Cell* 2004, **15:**706-720.
[6] Mollapour M, Fong D, Balakrishnan K, Harris N, Thompson S, Schüller C, Kuchler K, Piper PW: **Screening the yeast deletant mutant collection for hypersensitivity and hyper-resistance to sorbate, a weak organic acid food preservative.** *Yeast* 2004, **21:**927-946.
[7] Simões T, Teixeira MC, Fernandes AR, Sá-Correia I: **Adaptation of *Saccharomyces cerevisiae* to the herbicide 2,4-dichlorophenoxyacetic acid, mediated by Msn2p- and Msn4p-regulated genes: important role of *SPI1*.** *Appl Environ Microbiol* 2003, **69:**4019-4028.
