# Supplementary material for: Search for genes responsible for the remarkably high acetic acid tolerance of a Zygosaccharomyces bailii-derived interspecies hybrid strain
Source: BMC Genomics. 2015 Dec 16;16:1070. doi: 10.1186/s12864-015-2278-6 (PMC4681151; doi:10.1186/s12864-015-2278-6)
Supplement: Additional file 3: — Partial sequence of the 26S ribosomal RNA gene of Z. bailii strain IST302. (PDF 4 kb) [file 12864_2015_2278_MOESM3_ESM.pdf]

>*Z. bailii* IST302_ 26S ribosomal DNA gene, partial sequence

GAAAAGAAACCAACCGGGATTGCCTTAGTAACGGCGAGTGAAGCGGCAAAAGCTC
AAATTTGAAATCTGGTACCTTCGGTGCCCGAGTTGTAATTTGTAGAAGGCGACTCTG
GGGCTGGTCCTTGTCTATGTTCCTTGGAACAGGACGTCATGGAGGGTGAGAATCCC
GTATGGCGAGGATCCCAGTTCTTTGTAGAGTGCCTTCGAAGAGTCGAGTTGTTTGG
GAATGCAGCTCTAAGTGGGTGGTAAATTCCATCTAAAGCTAAATATTGGCGAGAGA
CCGATAGCGAACAAGTACAGTGATGGAAAGATGAAAAGAACTTTGAAAAGAGAGT
GAAAAAGTACGTGAAATTGTTGAAAGGGAAGGGCATTTGATCAGACATGGTGTTTT
GCGCCCCTCGCCTCTCGTGGGTGGGGGAATCTCGCAGTTCACTGGGCCAGCATCAG
TTTTGGCGGCAGGATAAATCCCTGGGAATGTAGCTCTACCACTTCGTGGCGGACGA
ACTTATAGTCCAGGGGAATACTGCCAGCTGGGACTGAGGAATGCGACTTTTAGTCA
AGGATGCTGGCATAATGGTTATATGCCGCCCGTCTTGAAACACGGACC
