# Supplementary material for: Search for genes responsible for the remarkably high acetic acid tolerance of a Zygosaccharomyces bailii-derived interspecies hybrid strain
Source: BMC Genomics. 2015 Dec 16;16:1070. doi: 10.1186/s12864-015-2278-6 (PMC4681151; doi:10.1186/s12864-015-2278-6)
Supplement: Additional file 4: — List of primers used in this work. All the primers contain a region with homology to the gene/region to be amplified (italics) and a nucleotide sequence with homology to the cloning site flanking region of the vector used for recombination (underlined). (PDF 170 kb) [file 12864_2015_2278_MOESM4_ESM.pdf]

Primer	Sequence (5’-3’)	Amplified Product	Vector used for recombination of the amplified product
ZBAI_02295_FWD	GAATTCGATATCAAGCTTATCGATACCGTCGACA*ATGTATTTTGTGGATTGCATGGAG*	ZBAI_02295	pGREG506
ZBAI_02295_REV	GCGTGACATAACTAATTACATGACTCGAGGTCGAC*TCAGTTGAAAATGATATCATAAATC*		
ZBAI_02296_FWD	GAATTCGATATCAAGCTTATCGATACCGTCGACA*ATGACACGAAGTGACAGCGCTTCA*	ZBAI_02296	pGREG506
ZBAI_02296_REV	GCGTGACATAACTAATTACATGACTCGAGGTCGAC*CTATGGAAAGTTATGAATTGAAGG*		
ZbMSN4_pGREG_FWD	GAATTCGATATCAAGCTTATCGATACCGTCGACA*ATGAGTGATCTCTTCATGGAGTTG*	*ZbMSN4*	pGREG506
ZbMSN4_pGREG_REV	GCGTGACATAACTAATTACATGACTCGAGGTCGAC*TTAAAAATCGCCGTGCTTCTTG*		
ZbMSN4prom_FWD	AACAAAAGCTGGAGCTCGTTTAAACGGCGCGCC*CAGGACCTCTGAAGGCAAAG*	*S. cerevisiae MSN4* promoter	pGREG506_ZbMSN4
ZbMSN4prom_REV	GTATCGATAAGCTTGATATCGAATTC*TATATTGTTTTTAATAAGAAGAAAAGAAAAAAAAG*		
ZbMSN4_FWD	TTTCCCCGAAAAGTGCCACCTGGTATCGGA*CAAGAGCATAGTTAACGCCGAC*	*ZbMSN4* + 1000bp upstream the coding region	pZ$_3$*b*T
ZbMSN4_REV	GGAAAAACGTTCATTGTTCCTTATTCAGTTAG*TTAAAAATCGCCGTGCTTCTTG*		
ZbTIF3_FWD	TTTCCCCGAAAAGTGCCACCTGGTATCGGA*ACA ACC TCT ATC CTG AAC GG*	*ZbTIF3* + 1000bp upstream the coding region	pZ$_3$*b*T
ZbTIF3_REV	GGAAAAACGTTCATTGTTCCTTATTCAGTTAG*TTACTTTTTGCCCACAACATTCC*		
